# Heterotopic and Orthotopic Tracheal Transplantation in Mice used as Models to Study the Development of Obliterative Airway Disease

**DOI:** 10.3791/1437

**Published:** 2010-01-20

**Authors:** Xiaoqin Hua, Tobias Deuse, Karis R. Tang-Quan, Robert C. Robbins, Hermann Reichenspurner, Sonja Schrepfer

**Affiliations:** Transplant and Stem Cell Immunobiology Lab (TSI), University Heart Center Hamburg; CVRC, University Hospital Hamburg; Department of CT Surgery, Stanford University School of Medicine

## Abstract

Obliterative airway disease (OAD) is the major complication after lung transplantations that limits long term survival (1-7).

To study the pathophysiology, treatment and prevention of OAD, different animal models of tracheal transplantation in rodents have been developed (1-7). Here, we use two established models of trachea transplantation, the heterotopic and orthotopic model and demonstrate their advantages and limitations.

For the heterotopic model, the donor trachea is wrapped into the greater omentum of the recipient, whereas the donor trachea is anastomosed by end-to-end anastomosis in the orthotopic model.

In both models, the development of obliterative lesions histological similar to clinical OAD has been demonstrated (1-7).

This video shows how to perform both, the heterotopic as well as the orthotopic tracheal transplantation technique in mice, and compares the time course of OAD development in both models using histology.

**Figure:**
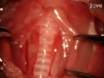


## Protocol

Male Balb/C mice (8-12 weeks) are purchased from Charles River Laboratories (Sulzfeld, Germany). The mice are housed under conventional conditions, fed standard mice food and water ad libitum.
2% Isofluran is used for anesthesia.

### DONOR PREPARATION

Shave the abdominal hair and disinfect the area using betaisodona.
Under microscopic view, perform a midline cervical incision from the level of the larynx to the sternum.
Remove the subcutaneous fat and the strap muscles to get a clear view of the trachea.
Dissect the trachea from any surrounding tissues, like the esophagus, nerves, arteries, and connective tissue.
Remove the whole trachea (from the larynx to the bifurcation).
Flush the transplant with cold saline and store the graft at 4°C.
The donor is euthanized by cervical dislocation following the harvest of the trachea.

### RECIPIENT: HETEROTOPIC TRANSPLANTATION

Shave the abdominal hair in a wide margin around the incision site and disinfect the area three times using betaisodona (betadine) followed by alcohol. Eyes should be lubricated with an ophthalmic ointment product to prevent the corneas from drying out.
Perform a median laparotomy and place the intestine into a sterile, moistured glove.
Spread the greater omentum carefully. Place the graft into the center and fixate it with a single suture using 8-0 (Prolene, Ethicon, Germany).
Fully cover the transplant with the greater omentum and fix the graft with one single suture 8-0 (Prolene, Ethicon, Germany).
Relocate the intestines back into the abdomen and flush with warm, sterile saline prior to closure.
Close up in 2 layers - abdominal wall and skin layer with continuous pattern using 7-0 Prolene for the muscle and 7-0 Vicryl for the skin.

### RECIPIENT: ORTHOTOPIC TRANSPLANTATION

Shave the abdominal hair in a wide margin around the incision site and disinfect the area three times using betaisodona (betadine) followed by alcohol. Eyes should be lubricated with an ophthalmic ointment product to prevent the corneas from drying out.
Divide the strap muscles to visualize the entire laryngotracheal complex.
Carefully dissect the trachea from the surrounding tissues, take care to preserve the recurrent laryngeal nerves.
Divide the trachea three rings caudal from the cricoid. The animal maintains physiologic respiration via the tracheostomy.
Ensure clean tracheal edges in the recipient as well as in the graft.
The graft is interposed between the recipient tracheal defects and orientated to maintain anatomic polarity.
Using 8-0 (Prolene, Ethicon, Germany) anastomose the donor graft with the distal (mediastinal) trachea. The posterior aspect of the anastomosis is performed in continuous running fashion. The anterior aspect is then completed using interrupted stitches.
Remove any secretions from the airway.
The proximal anastomosis is then completed in the same way as the distal one.
Ensure integrity of the airway and adequate, spontaneous breathing.
Relocate the strap muscles and close the subcutaneous tissue and skin layer using 6-0 sutures (Vicryl, Ethicon, Germany) with continuous pattern.
Use injection anesthesia for the recipient, therefore the animal will retain physiological respiration through the trachea. A combination of 75/1/0.2 mg/kg of propofol, medetomidine and fentanyl, respectively, is used for i.p. anaesthesia in mice.

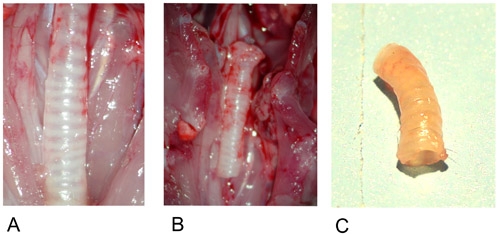
 **Figure 1: Donor trachea. **1A: Donor trachea in situ after preparation. 1B: Excised donor trachea. 1C: Donor trachea after explantation.


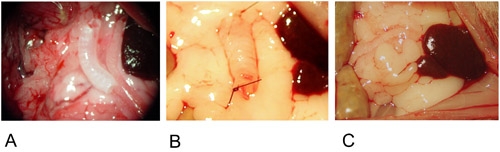
 **Figure 2: Heterotopic Model.**2A: The graft is positioned in the center of the greater omentum. 2B: The graft is fixed on both ends with a single suture. 2C: The graft is wrapped into the greater omentum and fixed with a single suture.


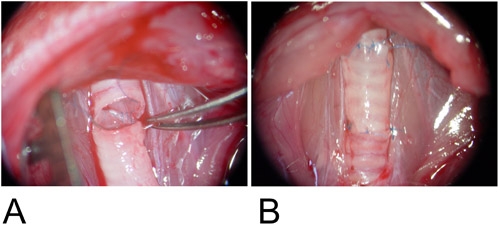
 **Figure 3: Orthotopic Model.**3A: The graft is interposed between the recipient tracheal defects and the posterior wall is anastomosed in continuous running fashion. 3B: The anterior wall is completed using a single suture.


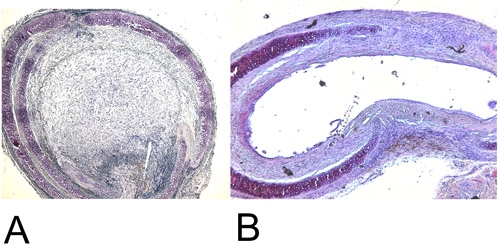
 **Figure 4: Histology.**4A: Recovered heterotopic transplanted trachea after 28 days in the H+E staining (15x). Note the 100% luminal obliteration. 4B: Recovered orthotopic transplanted trachea after 60 days in the H+E staining (15x). Maximal achieved luminal obliteration is approximately 45%.

**Table:** 

	**Heterotopic Tracheal Transplantation Model**	**Orthotopic Tracheal Transplantation Model**
**Advantages**	+ Easy to perform + Luminal obliteration with complete airway occlusion after 28 days + No physical affection of animals by OAD	+ Physical ventilation of the graft + Inhaled drug administration possible + Strong immunological reactions such as alloreactive IgM antibody production + Physiological thoracic milieu + Tracheal-tracheal anastomosis imitates the clinical setting
**Disadvantages**	- No ventilation of transplanted trachea - No evaluation of inhaled pathogens possible - Inhibition of mucociliary clearance and retained secretions - Peritoneal microenvironment instead of thoracic milieu	- Surgical training necessary - Luminal obliteration with luminal occlusion app. 45% after 60 days - Animals may develop symptoms of OAD

**Table 1:** Advantages and Disadvantages of Heterotopic and Orthotopic Tracheal Transplantation.

## Discussion

Mice are available in different transgeneic and knockout model, and therefore suitable to study mechanistic questions related to OAD (4). Both models of tracheal transplantation shown in this video can be used as reliable models for studying OAD development. However, each model demonstrates advantages and limitations.

The **heterotopictracheal transplantation** is easy to perform and does not require special surgical training (3, 5). After heterotopic transplantation, luminal obliteration will occur fast and complete airway occlusion appears after 28 days (3, 4, 6). Animals are not physically affected by the OAD development, since their organism does not depend on the heterotopic transplanted trachea.

A disadvantage is the lack of ventilation of the transplanted trachea (7, 6), therefore it is not possible to evaluate the influence of inhaled pathogens (3, 7). Due to the inhibition of mucociliary clearance and retained secretions the results may differ from physiologic reactions seen in clinical OAD (7). The peritoneal microenvironment differs from the thoracic milieu which may also lead to altered results (3).

To perform the **orthotopic tracheal transplantation** surgical training is necessary and luminal obliteration of the transplanted trachea appears after 60 days instead of 28 days in the heterotopic model (3, 7). Also, maximal luminal obliteration achieved is app. 45% instead of 100% in the heterotopic model (3, 7). However, in the orthotopic model, physiologic ventilation is obtained and inhaled drug administration is possible (3). Immunological response, such as alloreactive IgM antibody production has been shown to be much stronger in this model than the heterotopic model (3). The tracheal-tracheal anastomosis and the reactions in this site are more comparable with the anastomosis performed in the clinic (7).

Advantages and limitations of each model are shown in detail in Table 1.

In summary, this video shows that both, the heterotopic as well as the orthotopic tracheal transplantation technique in mice can be used as reproducible and reliable models to study OAD. 

However, the model should be chosen carefully depending on the basic question of the study.
